# Corrigendum: Controlling hybrid nonlinearities in transparent conducting oxides via two-colour excitation

**DOI:** 10.1038/ncomms16139

**Published:** 2017-08-01

**Authors:** M. Clerici, N. Kinsey, C. DeVault, J. Kim, E. G. Carnemolla, L. Caspani, A. Shaltout, D. Faccio, V. Shalaev, A. Boltasseva, M. Ferrera

Nature Communications 8 Article number: 15829 ; DOI: 10.1038/ncomms15829 (2017); Published 06 09 2017; Updated 08 01 2017

An incorrect version of the Supplementary Information was inadvertently published with this Article, where the pump wavelengths for panels a and b were interchanged in the figure caption of Supplementary Fig. 3. The HTML has now been updated to include the correct version of the Supplementary Information.

