# Supplementary material for: Controlling hybrid nonlinearities in transparent conducting oxides via two-colour excitation
Source: Nat Commun. 2017 Jun 9;8:15829. doi: 10.1038/ncomms15829 (PMC5472708; doi:10.1038/ncomms15829)
Supplement: Supplementary Information — Supplementary Notes, Supplementary Figures, Supplementary Discussion and Supplementary References [file ncomms15829-s1.pdf]

Supplementary Information

Supplementary Notes

Supplementary Note 1. Crosstalk between nonlinearities

Supplementary Figure 1 shows that the crosstalk between the effects of interband and intraband nonlinearities is minimal, thus allowing for an effective algebraic summation of the induced effects. However, we observed experimentally that this approximation is valid for pump fluences up to a certain value. We established this by an in-depth analysis of the interactions between interband and intraband nonlinearities at different pump fluences. The results are shown in Supplementary Figure 2. This analysis allowed us to quantitatively define the boundaries within which interband and intraband nonlinearities can be considered independent. A theoretical investigation of the nonlinear interaction between the two nonlinearities is the topic of an ongoing investigation.

Supplementary Note 2. Generality of the effects extended to other materials

It is reasonable to ask if the reported effects are specific of aluminium-doped zinc oxide (AZO) or shared among other transparent conductive oxide (TCO) materials. In this regard, we performed a preliminary comparison between commercially purchased indium tin oxide (ITO) films and the oxygen-deprived AZO films in terms of their transient relative transmissivity. The choice of ITO over other TCOs relies on its widespread use, which makes it the most important representative for the entire class of TCOs. The results for a 310 nm thick ITO film are reported in Supplementary Figure 3, which shows that both interband and intraband nonlinearities are available, and still with opposite effects on the probe beam transmission. While a comparison between the AZO and ITO nonlinearities is beyond the purpose of this work, some observations can be drawn from the current measurements, although performed on two films of different thickness (AZO = 900 nm, ITO = 310 nm). In the specific excitation conditions considered in our experiments, ITO shows a strong imbalance between intraband and interband effects, with the former having a magnitude more than twice the latter. In addition, the relaxation time upon ultraviolet (UV) excitation is much longer than the one we recorded for AZO (e.g. interband recombination time, $\tau_{\mathrm{rec}}^{\mathrm{AZO}} \simeq 600$ fs and $\tau_{\mathrm{rec}}^{\mathrm{ITO}} \simeq 260$ ps). In conclusion, our results hint at the possibility of performing effective two-colour excitation in TCOs other than AZO, although the latter boasts faster recombination times and stronger nonlinearities.

Supplementary Figures

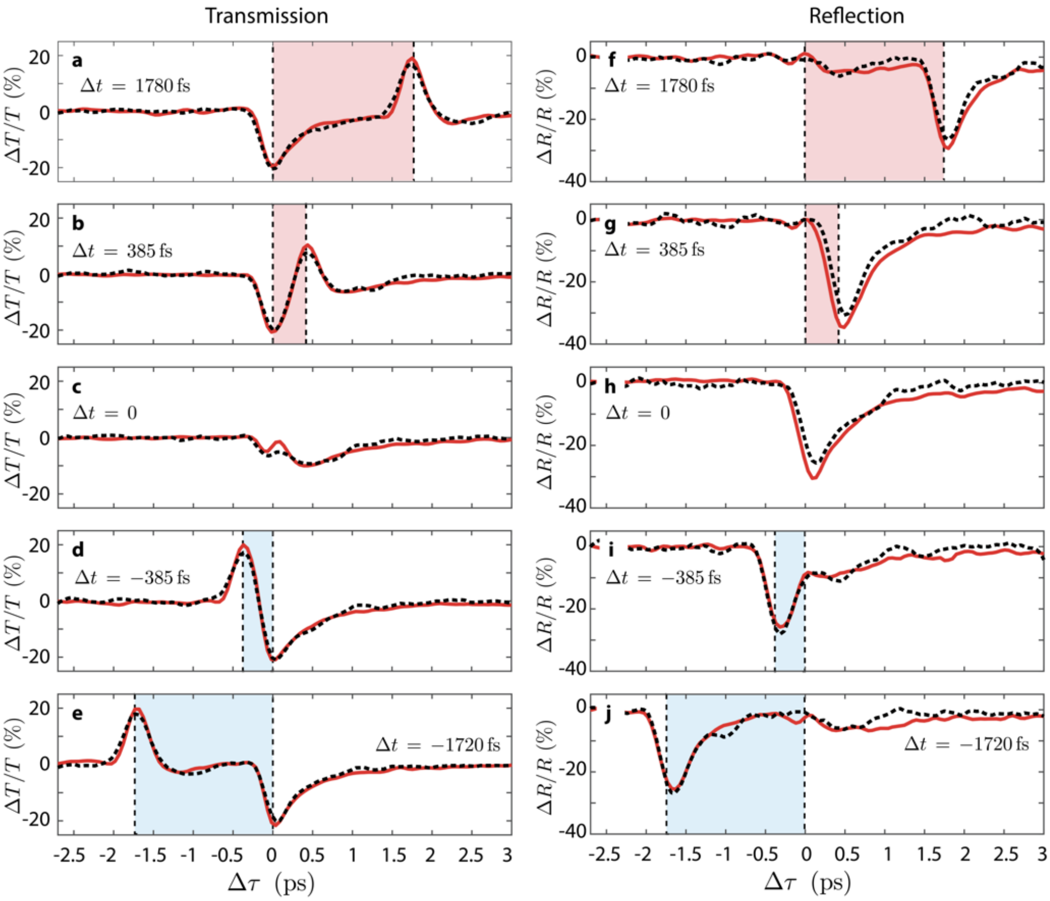

Supplementary Figure 1. Modulation of transmission and reflection for the AZO film. Transient transmissivity (**a-e**) and reflectivity (**f-j**) as a function of the time delay $\Delta\tau$ between the ultraviolet (UV) pump and the probe. The multiple graphs refer to different values of the time distance Δt between the UV and the near infrared (NIR) pump pulses. The shaded red and blue regions indicate positive and negative delays, respectively. For pump fluences used in these acquisitions ($F_{UV} \simeq 5\ mJ\ cm^{-2}$; $F_{NIR} \simeq 14\ mJ\ cm^{-2}$) modulations of the order of $\pm$ 20 % and 35 % were recorded for the transmitted and the reflected signal, respectively. In all cases, the overall dynamics (generation + recombination) is in the sub-picosecond region. A longer relaxation time was measured for the interband transitions ($\simeq 600\ fs$) than for the intraband case ($\simeq 170\ fs$). The optical modulation produced by the two effects is opposite in sign. When the two pumps were temporally overlapped, as in **c**, we recorded an almost complete cancellation of the nonlinear effects, mainly limited by the different relaxation times relative to the interband and intraband processes. We note that numerous measurements were performed for several probe wavelengths and incident pump powers to evaluate the optimal operational wavelength for enhancing nonlinearities. As expected from theory, the optimal spot fell in the proximity of the zero-epsilon wavelength (ENZ region) at λ =1308 nm. This probe wavelength was used through all the experiments shown in the figure.

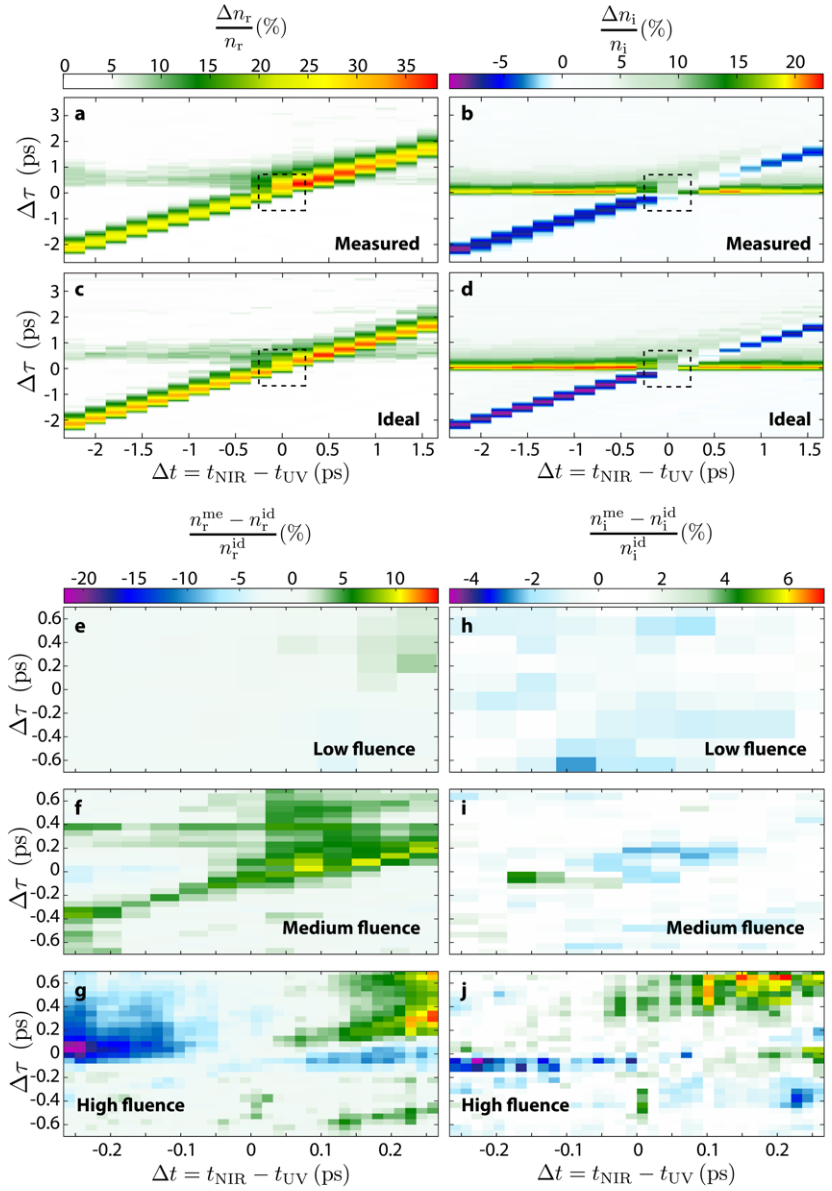

Supplementary Figure 2. Crosstalk between nonlinearities. (**a-d**) show the relative transient changes of the real (**a**, **c**) and imaginary (**b**, **d**) part of the refractive index as a function of the time delay $\Delta\tau$, between the ultraviolet (UV) pump and the probe, and Δt, between the UV and the near infrared (NIR) pump pulses. The label "measured" refers to those results dealing with the simultaneous interband and intraband excitations, while "ideal" indicates plots attained by the algebraic summation of the refractive index change induced by independent UV and IR pumping. **a** and **b** show the relative change in the real and imaginary refractive index, respectively, for pump fluences of $F_{\mathrm{UV}} = 5\ \mathrm{mJ\ cm^{-2}}$ and $F_{\mathrm{NIR}} = 14\ \mathrm{mJ\ cm^{-2}}$. **c** and **d** show the same quantities evaluated by the algebraic summation of the effects recorded with separate NIR and UV pumps. The amount of crosstalk between the two nonlinearities can be appreciated by comparing the correspondent "measured" (**a, b**) and "ideal" (**c, d**) plots in the time region where Δt and $\Delta\tau$ approach zero, identified by the black dashed box. At these pump fluences, which were employed for the experiments shown in the main manuscript, no significant crosstalk between the two nonlinearities is evident. To better evaluate the amount of crosstalk we measured the relative difference between the ideal change in the real (**e-g**) and imaginary (**h-j**) refractive index as a function of the two delays Δt and $\Delta\tau$ (in a region where they approach zero) for three different pump fluence conditions. We quantify the crosstalk plotting the quantity: $(n_{\mathrm{r,i}}^{\mathrm{me}} - n_{\mathrm{r,i}}^{\mathrm{id}})/n_{\mathrm{r,i}}^{\mathrm{id}}$, where r and i stand for real and imaginary, and me and id stand for measured and ideal. (**e**, **h**) were recorded at low pump fluences ($F_{\mathrm{UV}} = 1.6\ \mathrm{mJ\ cm^{-2}}$ and $F_{\mathrm{NIR}} = 9\ \mathrm{mJ\ cm^{-2}}$) and show negligible crosstalk ($< 6\ \%$ for n_{r}; and $< 3\ \%$ for n_{i}). (**f, i**) were recorded for the medium fluences used in the main experiment ($F_{\mathrm{UV}} = 5\ \mathrm{mJ\ cm^{-2}}$ and $F_{\mathrm{NIR}} = 14\ \mathrm{mJ\ cm^{-2}}$) and show the onset of limited crosstalk ($< 10\ \%$ for n_{r}; and $< 5\ \%$ for n_{i}). (**g, j**) were recorded at high pump fluences ($F_{\mathrm{UV}} = 10\ \mathrm{mJ\ cm^{-2}}$ and $F_{\mathrm{NIR}} = 24\ \mathrm{mJ\ cm^{-2}}$) and show appreciable crosstalk ($\simeq 22\ \%$ for n_{r}; and $\simeq 5\ \%$for n_{i}).

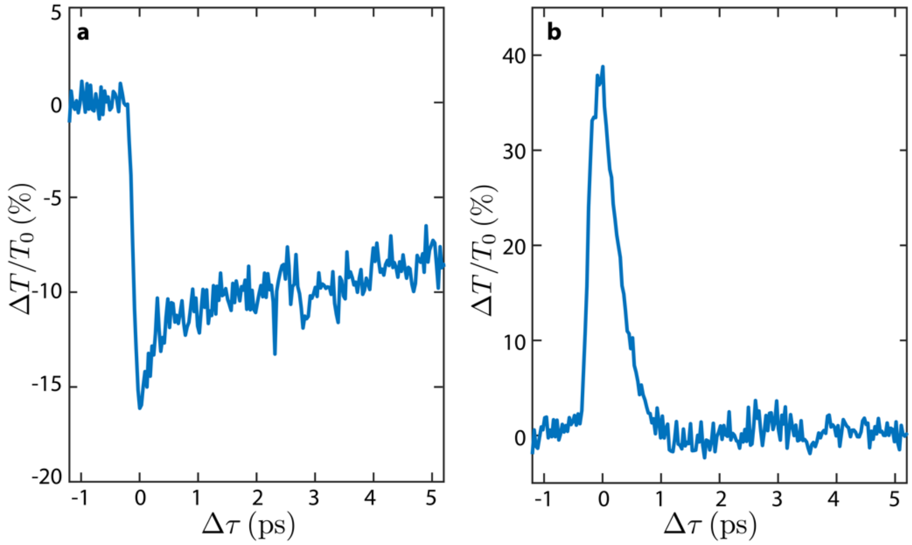

Supplementary Figure 3. Nonlinear effects with indium tin oxide (ITO) thin films. Relative transient transmissivity as a function of $\Delta\tau$ for (**a**) ultraviolet (UV, 262 nm) and (**b**) near-infrared (NIR, 787 nm) pumping of a 310 nm thick ITO film. The probe wavelength was set at the correspondent zero-epsilon point of the ITO film (1239 nm) to make a fair comparison with the results obtained with the aluminium-doped zinc-oxide films discussed in the main text. The data were acquired at pump fluences slightly below the damage threshold ($F_{\mathrm{UV}} = 22\ \mathrm{mJ\ cm^{-2}}$ and $F_{\mathrm{NIR}} = 42\ \mathrm{mJ\ cm^{-2}}$)

Supplementary Discussion

Interband dynamics and modelling. The interband modulation ($E_{\mathrm{h\nu}} > E_{\mathrm{g}}$) and relaxation of our AZO film were first observed individually using only the 262 nm UV pump and probe. In this situation, electron-hole pairs ($\delta_{\mathrm{n}}, \delta_{\mathrm{p}}$) are formed in the material which results in a change in the optical properties through the Drude formula, see Supplementary Equation 1, until they recombine (τ_{rec}). The sub-picosecond recombination time is indicative of Shockley-Read-Hall recombination processes which result in a reduced recombination time according to $\tau_{\mathrm{rec}} \propto 1/N_{\mathrm{t}}\sigma v_{\mathrm{th}}$ where N_{t} is the trap density, σ is the capture cross section, and v_{th} is the thermal velocity of carriers.[1] These mid-gap defect states are an interesting, and useful, byproduct of our unique growth procedure designed to enhance the intrinsic carrier density in the AZO material through Al doping and a severe oxygen deprivation.[2] This oxygen-deprived fabrication procedure is believed to be the primary cause for the ultra-fast recombination time related to the interband relaxation. Furthermore, the large relative change in the transmission is the result of operating in the epsilon-near-zero regime, whereby the refractive index of the material can be modulated on the order of the steady-state refractive index.[2,3]

The interband dynamics are modelled using a 2-D spatial and temporal discretization. The incident photon fluence is determined from the experimental conditions of the excitation pulse and after correction for reflection of the multilayer material, is propagated through the medium and the fluence per unit thickness δz into the material is calculated. The carrier density per unit δz is then determined as the difference between the fluences divided by δz. The populations of electrons and holes are

assumed equal. The temporal response of the pump-probe experiment is then applied to carrier density at each position z.[4] Thus, the final 2-D carrier density is given by:

$$\delta_{\mathrm{n,p}}(z,t) = e^{-\frac{t}{\tau_1}} \left[1 - erf\left(\frac{w}{2\,\tau_1} - \frac{t}{w}\right)\right] \sum_{m=0}^{n-1} (1-R)\, F_0 \left(\frac{e^{-\alpha\, z(m)} - e^{-\alpha\, z(m+1)}}{\delta z}\right) \quad (1)$$

where τ_1 is the recombination time, $w = \tau_{\mathrm{FWHM}}\,(2\ln 2)^{-1/2}$ is the cross-correlation width of the pump and probe pulses, R is the reflectivity of the un-pumped sample as determined by the transfer matrix method, α is the absorption coefficient, F_0 is the incident photon fluence, δz is the element size, n is the number of elements given by $n = \frac{t_{\mathrm{mat}}}{\delta z}$, where t_{mat} is the material thickness, and erf is the error function. The carrier density of both holes and electrons is then used to determine the change in the permittivity as given by:

$$\varepsilon(\omega) = \varepsilon_\infty + \frac{A\,\omega_0^2}{\omega_0^2 - \omega^2 - i\Gamma_0\omega} - \left(\frac{N_0+\delta_n}{m_{\mathrm{n}}^* m_0 \varepsilon_0}\right)\frac{q^2}{\omega^2 + i\Gamma_{\mathrm{n}}\omega} - \left(\frac{P_0+\delta_p}{m_{\mathrm{p}}^* m_0 \varepsilon_0}\right)\frac{q^2}{\omega^2 + i\Gamma_{\mathrm{p}}\omega} \quad (2)$$

where ε_∞ is the permittivity at high frequency, A is the strength of the Lorentz oscillator, ω_0 is the frequency of the Lorentz transition, Γ_0 is the loss factor of the Lorentz transition, N_0 and P_0 are the intrinsic electron and hole densities, m_{n}^* is the effective mass of electrons obtained from Hall measurements, m_{p}^* is the effective mass of holes, Γ_{n} and Γ_{p} are the loss factors of electrons and holes given by $\Gamma_{\mathrm{n,p}} = q\,\left(m_{\mathrm{n}}^* m_0 \mu_{\mathrm{n,p}}\right)^{-1}$, μ_{n} and μ_{p} are the carrier mobilities, q is the electron charge, m_0 is the electron mass, and ε_0 is the free-space permittivity. Due to the high intrinsic electron density, we take $P = P_0 + \delta_{\mathrm{p}} \simeq \delta_{\mathrm{p}}$. The effective mass and mobility of holes were taken from literature as $m_{\mathrm{p}}^* = 0.59$ [5] and $\mu_{\mathrm{p}} = 30\ \mathrm{cm}^2\ \mathrm{V}^{-1}\ \mathrm{s}^{-1}$ [6] for our films, although the inclusion of hole dynamics was observed to have a weak dependence upon the response of the system. Properties of the Lorentz oscillator and intrinsic carriers were captured from spectroscopic ellipsometry measurements as described in the Methods. Subsequently, the excess carrier density was calculated and assumed as a perturbation to this measured steady-state permittivity.

The change in the optical properties was then determined using the transfer matrix method for the graded index profile whereby a matrix was calculated for each layer δz and multiplied to determine an effective transfer matrix at each time step assuming an infinite substrate of fused silica. The amplitude of the change was then normalized and the recombination rate was fitted to the experimental data. Subsequently, the recombination time of the film was estimated to be $\simeq 600$ fs. The discrepancy in the recombination time with respect to previously reported values ($\simeq 100\ fs$) can be attributed to the different thickness of the samples under analysis and the subsequent effect on the defect density.[2] Specifically, the 900 nm sample used here is noted to have an intrinsic carrier concentration roughly double that of the previous work.[2] This is believed to be due to a reduction in the defect concentration

within the film and should occur on a similar scale to the change in carrier concentration. Since the recombination is primarily due to a defect enhanced processes,[2] the recombination time is subsequently expected to be increased by a similar factor. Although a similar method was used previously to fit both the temporal response and amplitude of the experiment,[2] the model predicts changes in the optical properties approximately 4 times larger than observed in the experiment. This discrepancy is believed to be due to a combination of several effects, such as band filling, bandgap renormalization and nonlinear absorption, which are important due to the small skin depth of the pump at 262 nm (i.e. $\simeq$ 50 nm). However, detailed density of states information is required to couple such effects to changes in the complex refractive index and is not available for our material. Previously, this was not important for our experiments as the carrier density was only slightly modified (i.e. $\simeq$ 10 % change)[2] while in this experiment it is predicted that the carrier density near the surface is more than doubled for a similar incident photon fluence. Future numerical and experimental efforts will be directed to better understand the density of states in this material to enable a more accurate modelling of the amplitude dynamics in AZO.

Intraband dynamics and modelling. The intraband dynamics ($E_{\text{hv}} < E_{\text{g}}$) of the AZO film were also observed individually using only the 787 nm excitation and probe beam. At this wavelength, the AZO is a lossy dielectric, but the excitation is still far from the band-edge ($\lambda \simeq$ 320 nm). Subsequently, the absorption in this regime is dominated by the residual Drude loss (i.e. free carriers in the conduction band). This excitation results in a non-equilibrium hot electron population which relaxes through various scattering processes ($\tau_{\text{e-p}}$), heating the lattice. The intraband dynamics of the AZO film are modelled using the two-temperature model, whereby the change in the electron temperature and lattice temperature are captured as a function of time for the material. Generally, the response is described by:

$$C_{\text{e}}(T_{\text{e}})\frac{\partial T_{\text{e}}}{\partial t} = \frac{\partial}{\partial x}\left(\kappa \frac{\partial T_{\text{e}}}{\partial x}\right) - G(T_{\text{e}} - T_{\text{l}}) + H \quad (3)$$

$$C_{\text{l}}(T_{\text{l}})\frac{\partial T_{l}}{\partial t} = G(T_{\text{e}} - T_{\text{l}}) \quad (4)$$

where C_{e} is the volumetric heat capacity of electrons, C_{l} is the volumetric heat capacity of the lattice ($C_{\text{l}} = 2.8 \times 10^{6}\ \text{J m}^{-3}\text{K}^{-1}$)[7], κ is the thermal conductivity ($\kappa = 100\ \text{W m}^{-1}\text{K}^{-1}$)[7], T_{e} is the temperature of the electrons, T_{l} is the lattice temperature, G is the electron-phonon coupling factor, and H is the source term given Supplementary Equation 5[8]:

$$H(x,t) = 0.94\,\frac{1-R-T}{w\,(\delta+\delta_{\text{b}})}\,J\,\frac{\exp\left[-\frac{x}{\delta+\delta_{\text{b}}} - 2.77\left(\frac{t}{w}\right)^{2}\right]}{1-\exp\left[-\frac{d}{\delta+\delta_{\text{b}}}\right]}. \quad (5)$$

Here, R is the reflectivity of the sample, T is the transmissivity of the sample, J is the excitation

intensity, δ is the skin depth of the pump, δ_b is the ballistic range of electrons, w the pump-probe cross-correlation width, and d is the sample thickness.

The coupled equations were solved numerically for both space and time assuming a ballistic range of hot electrons of 1 nm. Typically for gold, this is set to be $\simeq 100$ nm. However, in our film with many defects and grain boundaries, it is expected that the ballistic range is quite small for AZO.

The electron heat capacity was calculated as described in Kittel[9]

$$C_\mathrm{e}(T) = \frac{1}{2}\,\pi^2 N\,k_\mathrm{B}\frac{T}{T_\mathrm{F}}, \qquad (6)$$

where N is the intrinsic electron density ($N = 1{\times}10^{21}\ \mathrm{cm}^{-3}$), k_B is the Boltzmann constant, T is the equilibrium temperature, and T_F is the Fermi temperature (1.0 eV) above the conduction band minimum as obtained from Catellani *et al.*[10] This formula assumes that each defect or donor site contributes only one free electron. Subsequently, a value of $C_\mathrm{e} = 3500\ \mathrm{J\,m^{-3}\,K^{-1}}$ was determined. Following, the resulting change in the optical properties was modelled using an effective thermally dependent complex index, n_th, such that $\Delta n_\mathrm{AZO} = (\Delta T_\mathrm{e} + \Delta T_\mathrm{l})\,n_\mathrm{th}$. The transfer matrix method was used to determine the change in the optical properties of the graded index material (as described above). The reflection and transmission of the sample were normalized and the rate was fitted to extract the electron phonon-coupling coefficient and found to be $G \simeq 14\times10^{15}\ \mathrm{W\,m^{-3}\,K^{-1}}$. After the normalization procedure, only the sign of the complex effective thermal index is relevant, and it was found that the extinction coefficient decreased while the index increased (i.e. $n_\mathrm{th} > 0$ and $k_\mathrm{th} < 0$), matching the effect observed in experiments.

Supplementary References

1. Neamen, D. A. *Semiconductor physics and devices,* 4th edn. (McGraw-Hill Education, 2003).
2. Kinsey, N. *et al.* Epsilon-near-zero Al-doped ZnO for ultrafast switching at telecom wavelengths. *Optica* **2,** 616–622 (2015).
3. Babicheva, V. E., Boltasseva, A. & Lavrinenko, A. V. Transparent conducting oxides for electro-optical plasmonic modulators. *Nanophotonics* **4,** 165–185 (2015).
4. Prasankumar, R. P. & Taylor, A. J. *Optical Techniques for Solid-State Materials Characterization*. (CRC Press, 2011).
5. Jagadish, C. & Pearton, S. J. *Zinc Oxide Bulk, Thin Films and Nanostructures*. (Elsevier, 2011).
6. Ryu, Y. R., Lee, T. S. & White, H. W. Properties of arsenic-doped p-type ZnO grown by hybrid beam deposition. *Appl. Phys. Lett.* **83,** 87 (2003).
7. Morkoç, H. & Özgür, Ü. *Zinc Oxide: Fundamentals, Materials and Device Technology*. (Wiley-VCH, 2009).

8. Hohlfeld, J. *et al.* Electron and lattice dynamics following optical excitation of metals. *Chem. Phys.* **251,** 237–258 (2000).
9. Kittel. *Introduction to Solid State Physics,* 8th edn. (John Wiley and Sons, 2005).
10. Catellani, A., Ruini, A. & Calzolari, A. Optoelectronic properties and color chemistry of native point defects in Al:ZnO transparent conductive oxide. *J. Mater. Chem. C* **3,** 8419–8424 (2015).
